# Vertical dynamic patterns of *Vibrio* spp. in the northwestern Pacific Ocean

**DOI:** 10.3389/fmicb.2025.1649301

**Published:** 2025-09-08

**Authors:** Leihaothabam Jeeny, Keyi Huang, Xing Chen, Yan Wang, Shaodong Zhu, Yulin Zhang, Xiao-Hua Zhang, Xiaolei Wang

**Affiliations:** ^1^Frontiers Science Center for Deep Ocean Multispheres and Earth System and College of Marine Life Sciences, Ocean University of China, Qingdao, China; ^2^Laboratory for Marine Ecology and Environmental Science, Qingdao Marine Science and Technology Center, Qingdao, China; ^3^Key Laboratory of Evolution and Marine Biodiversity (Ministry of Education), Institute of Evolution and Marine Biodiversity, Ocean University of China, Qingdao, China

**Keywords:** *Vibrio* community, vertical distribution, environmental effects, assembly processes, Pacific Ocean

## Abstract

In marine ecosystems, *Vibrio* species are as they facilitate nutrient cycling and impact the condition of marine life. To understand their ecological dynamics and how they adapt to various environmental situations, this study examined the vertical distribution pattern and assembly processes of *Vibrio* species across a depth gradient (5–6000 m) within the Kuroshio Extension in the Northwest Pacific Ocean. Through quantitative PCR and high-throughput sequencing based on 16S rRNA genes, the abundance of *Vibrio* spp. showed a strong vertical stratification. *Vibrio* community compositions varied significantly among the ocean surface mixed layer (5-105 m, UL), the pycnocline and North Pacific Intermediate Water layer (155-700 m, ML), and bathypelagic layer (>1000 m, BL), which was reflected by a strong vertical depth decay pattern. In the UL, *Vibrio sagamiensis, Paraphotobacterium marinum, V. caribbeanicus, V. campbellii* and *Photobacterium phosphoreum* were the dominated species. *V. pomeroyi* was the most abundant species in ML and BL, and *V. sagamiensis, P. marinum* and *P. phosphoreum* usually persisted in deeper water layers, reflecting their potential adaptations to deep ocean conditions. Both deterministic factors (e.g., temperature, salinity, dissolved oxygen, ${\text{NO}}_{3}^{-}$, ${\text{PO}}_{4}^{3-}$ and ${\text{SiO}}_{3}^{2-}$) and stochastic processes shaped *Vibrio* community assembly mechanism, with stochasticity dominating community structure in UL and heterogeneous selection playing a key role in ML and BL. Our findings highlight the complex interplay between environmental gradients and stochasticity in shaping *Vibrio* communities along the depth in the water column, contributing to a deeper understanding of their dynamics in the open ocean.

## Introduction

*Vibrio* spp., belonging to the Gammaproteobacteria class, are famous microbes worldwide because several species are known human and animal pathogens, such as *V. cholerae, V. parahaemolyticus, V. vulnificus, V. anguillarum*, and *V. harveyi* (Siboni et al., 2016; Baker-Austin et al., 2018). For example, *V. vulnificus*, a common human infection in aquatic ecosystems like estuaries and marine shorelines, has caused public health and nutrition risks (Williams et al., 2022). Research has reported that *V. parahaemolyticus*, a pathogenic species causing gastroenteritis in humans, has diversified into four distinct groups, with notable convergence in their dissemination, possibly due to increased long-distance spread influenced by human activities like shipping and trade (Onohuean et al., 2022). Over the past few decades, the populations of *V. parahaemolyticus* have undergone significant genetic mixing, facilitated by their free dispersal across vast areas and habitats (Martinez-Urtaza et al., 2012).

Though pathogenic vibrios are hazards for coastal systems, there are more than 140 valid species (https://lpsn.dsmz.de/genus/vibrio) within the genus *Vibrio* and most of them are harmless (Zhang et al., 2018). *Vibrio* population have been considered as a low-abundance constituent in microbial assemblages, because they generally represent ~1% of the total bacterioplankton in most sea areas (Thompson et al., 2004b; Zhang et al., 2018; Wang et al., 2020b). Nevertheless, they are the important participators in nutrient cycling (especially the organic matter decomposition) and the overall functioning of aquatic food webs (Takemura et al., 2014; Zhang et al., 2018). *Vibrio* species have relatively short generation time and enable to have a broad metabolic range due to their highly plastic genomes (Takemura et al., 2014; Zhang et al., 2018), allowing them to rapidly response to nutrients plus like phytoplankton and inorganic (e.g., iron) bloom (Baffone et al., 2006; Takemura et al., 2014; Westrich et al., 2016). It has been reported that vibrios can consume a wide range array of organic carbon compounds as carbon and energy sources, with most species being able to degrade over forty species of compounds (Corzett et al., 2018; Zhang et al., 2018). A large amount of extracellular hydrolytic enzymes that can utilize polysaccharides (e.g., chitinase, agarase, laminarinase, and amylase) have been identified in vibrios (Sampaio et al., 2022; Zhang et al., 2022; Deng et al., 2025). Recently, several *Vibrio* species (e.g., *Vibrio gallaecicus*) have been found that they can convert methylphosphonate to methane (Wang et al., 2024; Yu et al., 2025). Together, these studies indicate that *Vibrio* spp. participate in the utilization and mineralization of carbon, nitrogen and phosphorus, highlighting their significant roles in the marine biogeochemical cycling (Takemura et al., 2014; Kopprio et al., 2017; Jesser and Noble, 2018).

Understanding the interaction of *Vibrio* community structures and dynamics is crucial for comprehending their ecological functions. The horizontal distribution of *Vibrio* spp. in estuarine and coastal environments worldwide has been well investigated, especially in the Chinese marginal seas (Zhang et al., 2018). Higher *Vibrio* abundance has been recorded in seawater and sediments of the Bohai Sea, Yellow Sea, East China Sea, and South China Sea compared to other sea areas worldwide (Wang et al., 2022). And, distinct *Vibrio* species varied among different areas, e.g., *Vibrio* sp. OTU13800 and *V. mimicus* in the Sydney Harbor estuary, *V. japonicus* and *V. harveyi* in the Ría de Vigo (Atlantic Ocean), and *V. atlanticus* and *V. owensii* in the Changjiang estuary, suggesting that all the local environments can be adapted by vibrios (Siboni et al., 2016; Liang et al., 2019; Wang et al., 2020b, 2022). In the vertical scale, *Vibrio* abundance and communities show significant differences across depths in the Yongle blue hole which has been divided into aerobic transition, middle anaerobic, and bottom anaerobic zones (Li et al., 2020a). In the eastern tropical Indian Ocean, *Vibrio* spp. exhibit notable lifestyle shifts, i.e., from free-living lifestyles on the surface seawater to mixed lifestyles at the bottom (Zhu et al., 2023). In contrast to marginal seas, the *Vibrio* community in the water column showed a markedly different structure, with significant vertical stratification in dominant species such as *Vibrio rotiferianus* mainly distributed in deeper water (Zhu et al., 2023). To the best of our knowledge, most studies have focused on coastal areas, often examining samples collected from a limited number of depths or within a narrow geographic range (Austin, 1988; Thompson et al., 2004a; Siboni et al., 2016; Zhu et al., 2023; Doni, 2024). Unstudied sea areas may harbor distinct *Vibrio* species due to local environmental conditions, and our understanding of the community dynamics and ecological roles of *Vibrio* spp. in the open ocean remains limited.

The distribution and composition of *Vibrio* communities across various environments can be significantly affected by stochastic processes and environmental factors, including temperature, salinity, and nutrient levels (Takemura et al., 2014; Johnson, 2015). And, previous studies have reported that temperature and salinity are the most common key factors, and other parameters like chlorophyll a (Chl a) and Dissolved Oxygen (DO) vary depending on the habitats (Takemura et al., 2014; Liang et al., 2019; Wang et al., 2020b). Recently, it has been found that both the deterministic (environmental factors) and stochastic processes have effects on *Vibrio* communities (Li et al., 2020b; Diner et al., 2021), and stochasticity usually govern the turnover of marine *Vibrio* communities at a small scale (e.g., the Beibu Gulf, China) (Shi et al., 2018; Li et al., 2020b). Vertical stratification influences microbial diversity and community distribution in ocean habitats by providing various ecological niches (Brown et al., 2022). The depth of the ocean significantly impacts the changes in environmental factors (Rogers, 2015). Usually, high temperatures, light availability and abundant nutrients support a more diverse and metabolic microbes in the surface layers (Naylor et al., 2022). As depth increases, temperature decreases, light availability diminishes and pressure rises leading to shifts in microbial composition (Hutchins and Fu, 2017). Nutrient concentrations may also vary, with organic matter sinking from surface waters providing a key resource for deeper microorganisms (Dahm et al., 1998). *Vibrio* spp. have been found in deeper and colder waters based on their abilities to adapt high pressures and low nutrients (Martinez-Urtaza et al., 2012; Zhu et al., 2023). For example, *Vibrio pomeroyi*, a species renowned for its ability to thrive in cold, nutrient-rich environments, is more prevalent in deeper waters (Lauro et al., 2009). Thus, in the Northwestern Pacific Ocean (NPO), where characterizes by great depths and exhibits highly dynamic environmental conditions, the change of vibrios from surface to bottom layers needs to be further studied.

The NPO exhibits highly dynamic vertical environments, driven by the interaction of major ocean currents such as the Kuroshio and Oyashio Currents. The Kuroshio Current exhibits characteristics of high temperatures, high salinity, and oligotrophy, whereas the Oyashio Current features low temperatures, low salinity, and high nutrient (Qiu, 2001; Fenies et al., 2023). Their convergence at the Kuroshio Extension (KE) makes it one of the most complex regions in global ocean dynamics (Qiu, 2001; Hu et al., 2024). From the surface down to the ocean bottom, the water column is structured into distinct layers by depth (Maximenko and Shcherbina, 1996). At the top is the ocean surface mixed layer (< 100 m), which enables the exchange of heat, momentum and dissolved gases between the atmosphere and the ocean (Johnson and Lyman, 2022; Roch et al., 2023). Between 100 and 200 m, it is the pycnocline where density changes most rapidly with depth is usually accompanied by sharp changes in both temperature and salinity (Sérazin et al., 2023). And, the North Pacific Intermediate Water layer (~300-800 m) which is the typical minimum salinity layer and relate to the mixed effect of Oyashio-Kuroshio (Liu et al., 2022), whereas the bathypelagic zone (>1000 m) which may be affected by North Pacific Deep Water (De Graaf et al., 2025). The intricate frontal structures, multiple water masses and mesoscale eddies in the NPO provided a unique environment for microbial community dynamics (Wang et al., 2020a). In this study, using quantitative PCR (qPCR) and high-throughput sequencing approaches, we investigated the vertical distribution pattern and community assembly of *Vibrio* spp. in the NPO, focusing on the relationship between *Vibrio* community dynamics and environmental gradients as well as ecological processes. We hypothesize that the abundance and distribution of *Vibrio* spp. may be diverse along the depths, affected by complex abiotic and biotic factors. Our results highlight the significance of considering stochasticity and environmental factors in understanding the community assembly of vibrios, enhancing the knowledge of their vertical distribution pattern in the open ocean.

## Materials and methods

### Sample collection and physicochemical parameter detection

**Figure 1:**
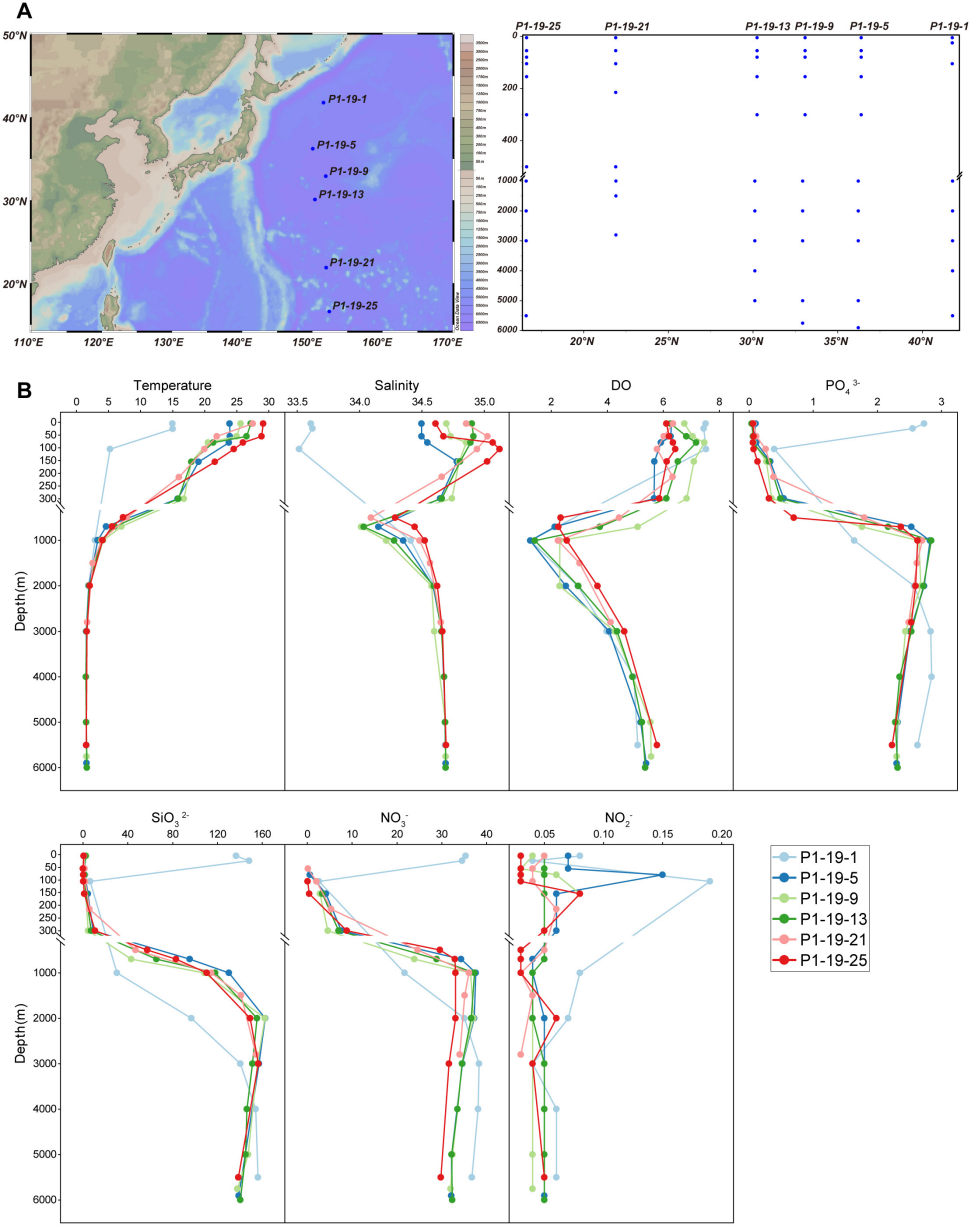
Description of sampling sites, vertical sample profile and environmental factors. **(A)** The sampling sites and their vertical profile that drawn by Ocean Data View [version 5.5.2; R. Schlitzer, Ocean Data View, https://odv.awi.de, 2021]). **(B)** Vertical distribution of significantly physicochemical parameters along depth.

Water samples were collected at six points along the P1 transect in NPO onboard the R/V *Dongfanghong 3* from October 31 to November 4, 2019 (Figure 1A). Comprehensive data for sampling sites was contained in the Supplementary Appendix Table 1. A total of sixty two seawater samples from six vertical sites (>8 samples per site) were collected using a 12-liter Niskin bottle and connected to an electroplating sampler with SeaBird CTD (SBE 911 model) to measure water depth, temperature, salinity, and Dissolved Oxygen (DO). All the seawater samples were divided into three groups, i.e., the ocean surface mixed layer (UL, 5-105 m; 19 samples), the pycnocline and North Pacific Intermediate Water layer (ML, 155-700 m; 15 samples), and the bathypelagic zone (BL, >1000 m; 28 samples). Approximately 1 L of seawater was filtered through 3μm and 0.22μm polycarbonate membranes (GTTP, 47 mm, Ispore) using a vacuum pump under low, non-disruptive pressure (< 5mm Hg). All filters were immediately frozen and stored in −80 °C onboard and transferred to a −80 °C freezer in the laboratory until DNA extraction. Samples for nutrients were collected, and the nutrients in each sample were measured based on the classical colorimetric method (Grasshoff et al., 2009), including ${\text{NO}}_{2}^{-}$, ${\text{NO}}_{3}^{-}$, ${\text{NH}}_{4}^{+}$, Dissolved Silicon (DSi) and Dissolved Inorganic Phosphorus (DIP). Water samples (500 ml) for Chlorophyll a (Chl a) analysis were filtered through a GF/F filter using a vacuum pump (< 10mmHg). Then, the filters were wrapped in aluminum foil and stored in the dark at −20 °C. They were extracted with 90% acetone and kept in the dark at 4 °C for 24 h, after which the concentrations of Chl *a* were determined by a Turner Designs Trilogy fluorometer (Parsons et al., 1984).

### DNA extraction

Using 3-μm and 0.22-μm polycarbonate membranes, DNA was extracted by the protocol described by Yin et al. (2013). Each polycarbonate membrane that seawater was filtered through was cut into pieces under sterilized conditions. A sterile tube was filled with 500 μl of sodium chloride-Tris-EDTA (STE buffer. Using FastPrep-24 homogenization equipment (MP Biomedicals, Irvine, California, U.S.A.), the solution was rapidly shaken twice to encourage cell lysis, resulting in a hydrolysis rate of 6.0 m/s. The DNeasy Power Water Kit (QIAGEN, U.S.A.) was then used to extract DNA according to the manufacturer's instructions. Then, the quantity and quality of the extracted DNA were detected by Nanodrop-2000 Spectrophotometer (ND-2000; Thermo Fisher Scientific), and the DNA samples were preserved at −80 °C until used.

### Quantitative PCR for total vibrios

16S rRNA gene-targeted qPCR was used to evaluate abundance of total *Vibrio* spp. Each DNA specimen was measured using the QuantStudio^TM^ 5 System (Applied Biosystems) and QuantStudio^TM^ Design and Evaluation Software. Specific 16S rRNA oligonucleotide primers for the genus *Vibrio*, V567F and V680R, were used in qPCR with SYBR-green detection (Thompson et al., 2004a; Vezzulli et al., 2015). The reaction system and conditions were performed according to Wang et al., 2022. The 16S rRNA genes of *Vibrio rotiferianus* WXL191, a species found in our lab, were used to create standard curves. According to Wang et al., 2022 technique each plate had standard curves and No-Template Control (NTC), in which ddH_2_O served in place of the template DNA. To ensure the reliability of the findings, each DNA sample conducted three rounds of qPCR analysis. The qPCR assay's amplification efficiency indicated an *R*^2^ value higher than 0.99, with values varied between 95% - 100%.

### High-throughput sequencing for *Vibrio* spp.

High-throughput sequencing aids in assessing microbial diversity in *Vibrio* species, using next-generation technologies like Illumina and Ion Torrent for population analysis, species identification, and ecological role investigation. *Vibrio-*specific primers V169F and V680R (Siboni et al., 2016) were amplified in the hypervariable regions of V2—V4 of the 16S rRNA gene to ascertain the general makeup of the *Vibrio* community. Using agarose gel electrophoresis, positive amplicons were verified. The PCR products were purified from 2% agarose gels using the AxyPrep DNA gel extraction kit (Axygen Biosciences, Union City, CA) and further quantified by QuantiFluor-ST (Promega) following the manufacturer's protocol. And, the amplicons were then pooled in equimolar and paired-end sequenced (2 × 300) on an Illumina Miseq PE300 platform at Majorbio Bio-Pharm Technology. Following the application of FLASH to combine raw fastq files, UPARSE (Version 11) was utilized to cluster operational taxonomic units (OTUs) at a 97% sequence similarity level (Gyraite et al., 2019; Zhu et al., 2023). The UCHIME programmed was employed to determine and dislodge chimeric sequences (Edgar et al., 2011). Using a minimum confidence level of 70%, the RDP classifier (Wang et al., 2007) was used to assign the taxonomy of each representative OTU 16S rRNA gene sequence against the SILVA 138 16S rRNA database (http://www.arb-silva.de). A more precise taxonomic identification was obtained by reassigning the *Vibrio* sequences to the EzBioCloud database (https://www.ezbiocloud.net/). With a “single rarefaction” QIIME script, sequences were subsampled based on the bare minimum of sample sequences for each sample to remove the impact of sampling effort on the analysis (Caporaso et al., 2010).

### Statistical analyses

To minimize biases associated with sequencing coverage, the number of sequences for each sample was homogenized to the lowest number (15,839 reads) by running a script in R software. Alpha diversity including Shannon and Simpson indices was calculated using the “vegan” package. The linear correlation between environmental parameters and α-diversity indices was performed using the “psych” package. For Beta diversity, the Principal Co-Ordinates Analysis (PCoA) was performed at the OTU level by using the “vegan” package. The subsequent Analysis Of Similarities (ANOSIM) was performed by using the anosim function with 999 permutations in “vegan”. The relationships between phylotypes and environmental factors were evaluated by db-RDA (distance-based Redundancy Analysis) in Canoco version 5.0. The analysis of the distance-decay pattern for the *Vibrio* spp. was conducted by using the function “vegdist” (“vegan” package), and Spearman's rank correlation test was used to test the significance of the correlations. The study correlated *Vibrio* abundance and environmental characteristics using Spearman's rank correlation analysis. To reveal the relationship between environmental factors and microbial communities, the Mantel test based on Pearson's correlations was carried out by the “ggcor” package. Additionally, a null model analysis was carried out to quantify the relative contributions of different ecological processes (Stegen et al., 2013), which was calculated using the “picante” package. The linear correlation between environmental parameters and βNTI was also performed by the “psych” package. Species with significant differences between groups were performed using STAMP (Parks et al., 2014).

## Results

### Environmental conditions

The environmental parameters of seawater collected from 6 sites in the NPO were measured (Figure 1B and Supplementary Table 1). The temperature gradually decreased along water columns from the surface (14-29 °C) to deep layers, and tended to stabilize in the BL (1-4 °C). The salinity of UL among all sites ranged from 33.6 (P1-19-1) to 34.9 PSU (P1-19-13), and increased rapidly until the maximum at 105 m, and then, it decreased slowly with depth, reaching the lowest value at 1,000 m. The Dissolved Oxygen concentration (DO) rapidly declined in the ML, and then slowly rebounded under 1,000 m. Additionally, the concentrations of ${\text{SiO}}_{3}^{2-}$, ${\text{NO}}_{3}^{-}$ and ${\text{PO}}_{4}^{4-}$ with depth showed consistent patterns. All these concentrations were relatively low in the UL, gradually increased with depth and stabilized at depths deeper than 1,000 m. The concentration of ${\text{NO}}_{2}^{-}$ was relatively stable along the whole depths, and fluctuated significantly within the ML. Interestingly, at site P1-19-1, the temperature and salinity of the UL and ML were significantly lower than those of other stations. Similarly, the variation patterns of the concentrations of ${\text{SiO}}_{3}^{2-}$, NO_3_^−^ and ${\text{PO}}_{4}^{3-}$ with depth in the water column at site P1-19-1 differed from other stations, and the concentrations in the UL are significantly higher than other samples.

### The abundance of total *Vibrio* spp.

**Figure 2:**
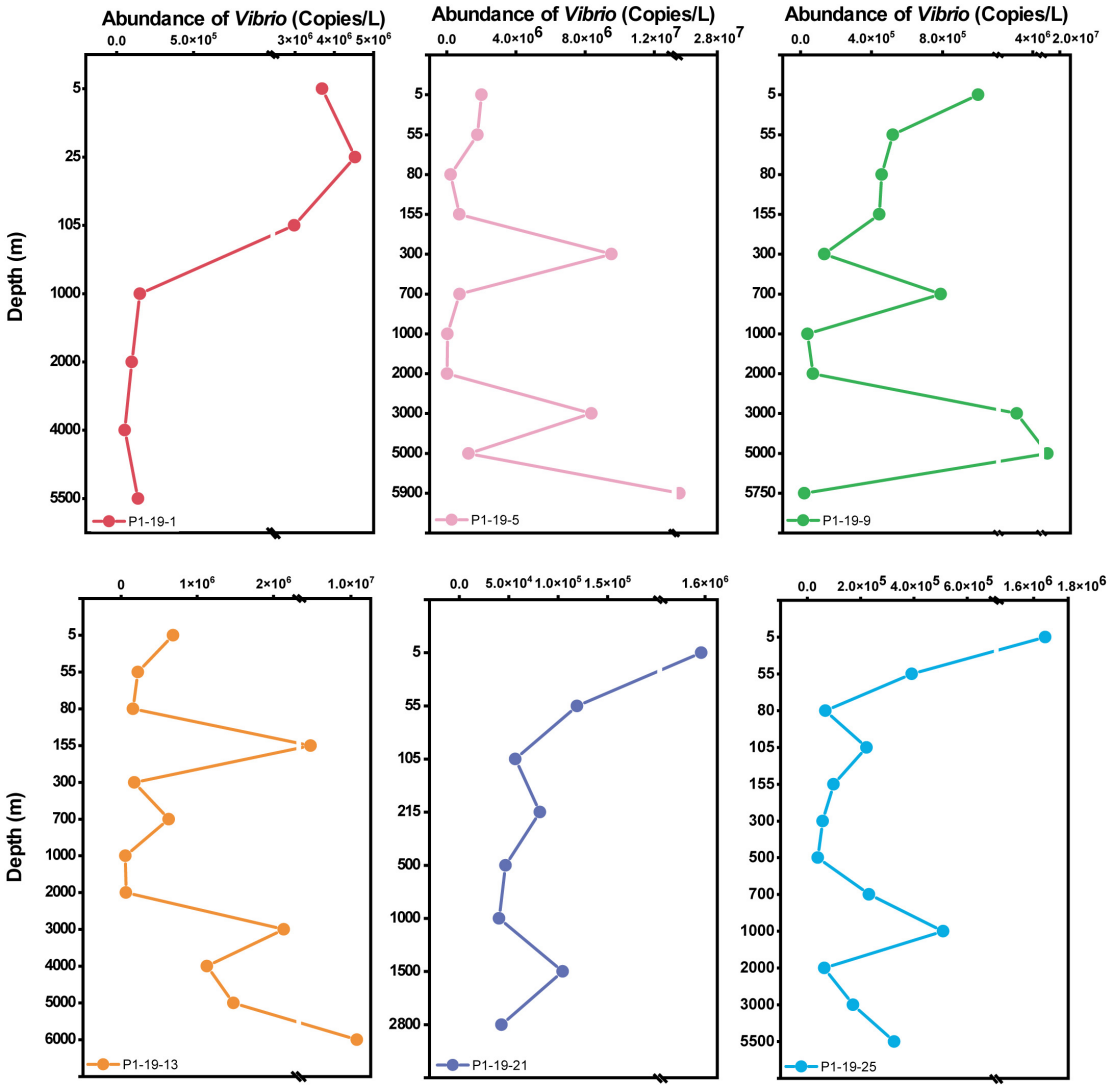
The vertical distribution of *Vibrio* abundance (copies/L) with depth at different sites.

The abundance of total *Vibrio* spp. in water columns among water layers was detected by qPCR (Figure 2). In general, the abundance of *Vibrio* at each site was ranged from 1.0 × 10^7^ ± 9.3 × 10^6^ to 3.9 × 10^4^ ± 1.5 × 10^4^ copies/L. At station P1-19-1, the abundance of *Vibrio* in the water column showed a decreasing trend with depth, from 3.7 × 10^6^ to 1.4 × 10^5^ copies/L. At stations P1-19-5, P1-19-9, and P1-19-13, the abundance of *Vibrio* fluctuated greatly in the water layers below 80 m (2.1 × 10^4^ to 2.4 × 10^7^ copies/L). As the sampling sites moved southward, i.e., at stations P1-19-21 and P1-19-25, the abundance of *Vibrio* spp. showed a decreasing trend with depth (from ~10^6^ to ~10^4^ copies/L) and was relatively stable in the BL.

### The diversity estimators of *Vibrio* spp.

**Figure 3:**
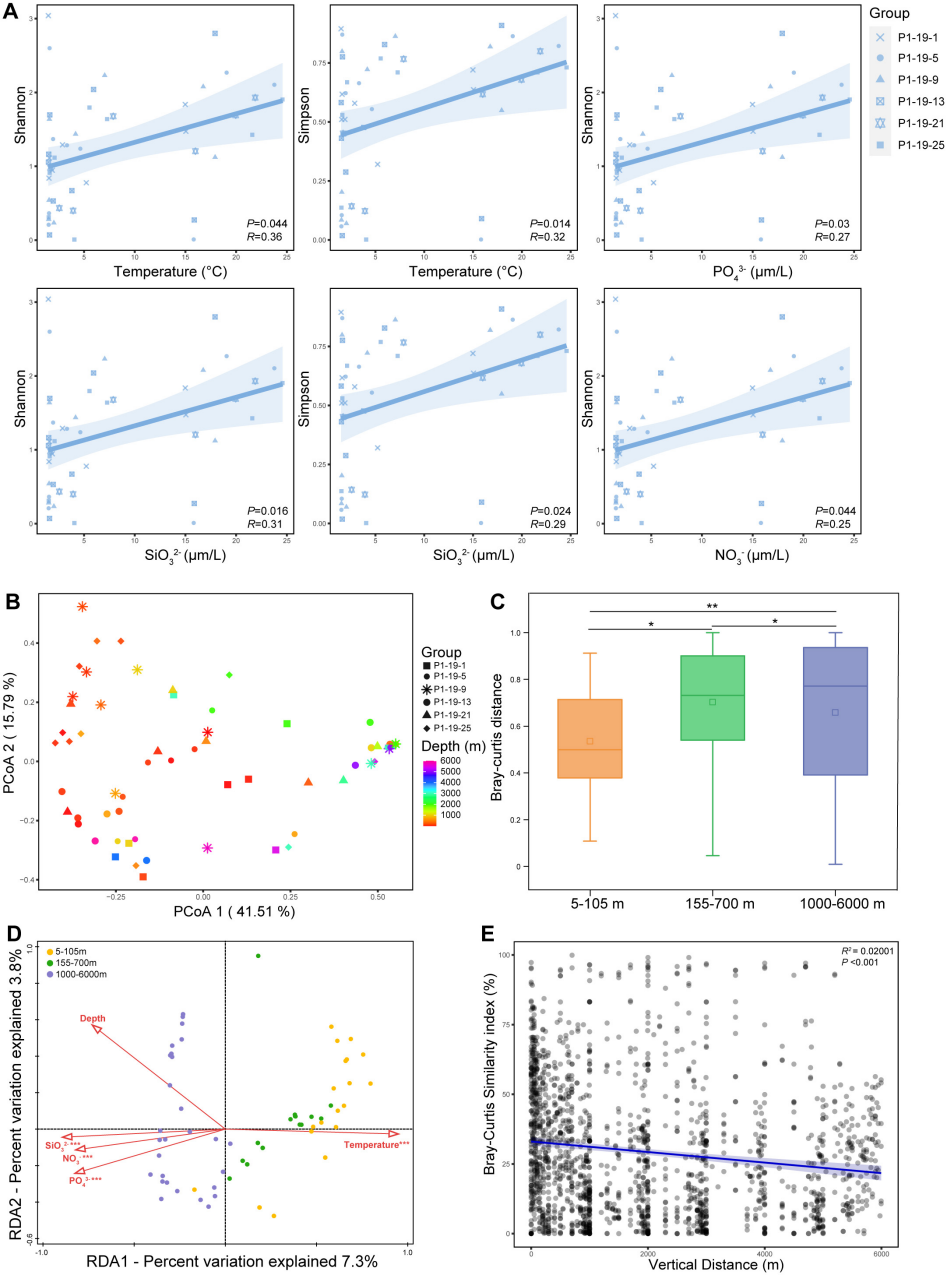
The alpha and beta diversity of *Vibrio* community at different sites. **(A)** The relationship between alpha index and main environmental factors. **(B)** Principal co-ordinates analysis (PCoA) of *Vibrio* community. **(C)** Bray-curtis distance of *Vibrio* community at 5-105 m, 155-700 m, and 1000-6000 m. ^*^P < 0.05; ^**^P < 0.01. **(D)** Redundancy Analysis (RDA) of *Vibrio* community. **(E)** The depth decay of Bray–Curtis similarity for *Vibrio* communities. Pairwise dissimilarities (the Bray-Curtis index) are plotted as a function of the distance among samples. The data are pairwise dissimilarities between the communities of all samples. The blue line represents the best linear regression result.

Nine lakh eighty two thousand hundred and eighteen high-quality reads were acquired after merging and filtering raw data for the 62 water samples. The total sequences yielded 611 Operational Taxonomic Units (OTUs) at a 97% sequence similarity. The sequencing coverages of all water samples were above 0.99, indicating that the retrieved sequences could represent most of the *Vibrio* community in the studied sites. The Shannon, Simpson, Chao 1, and Pielou's evenness indices were calculated to estimate α-diversity (Supplementary Table 2). Though no significant linear correlation was observed between the α-diversity indices and depth (*P* > 0.05), the community diversity (Shannon and Simpson) and evenness (Pielou's evenness) decreased from surface to ~3,000m layers and increased near the bottom (Supplementary Figure 1). And, the community richness (Chao 1 and Sobs) fluctuated along with depths (Supplementary Table 2). Regression analysis revealed that both Shannon and Simpson indices exhibit a positive correlation with temperature and ${\text{SiO}}_{3}^{2-}$, and Shannon index also positively correlated to ${\text{PO}}_{4}^{3-}$, and ${\text{NO}}_{3}^{-}$ (Figure 3A). As to the β-diversity, the results of Principal Co-ordinates Analysis (PCoA) indicated that total samples were divided into the UL, ML and BL and samples at similar depths were clustered together (Figure 3B). There were significant differences among the UL, ML and BL (ANOSIM, *P* < 0.05, Figure 3C). The relationship between the similarity of the *Vibrio* community and the vertical distance of the samples conformed to the distance-decay model (*P* < 0.001), and the community similarity decreased with the increase of depth (Figure 3E).

### Community compositions of vibrios

**Figure 4:**
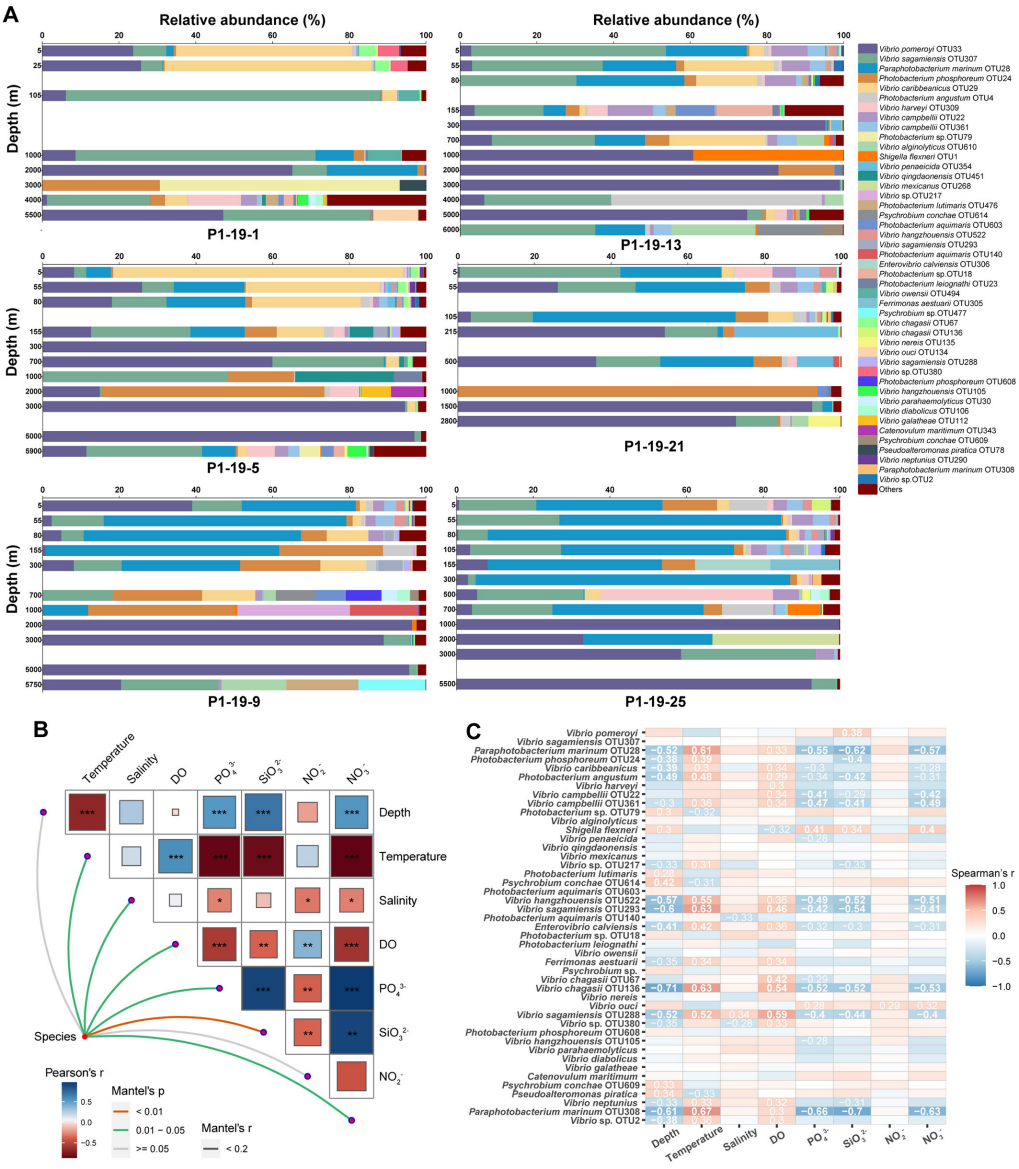
*Vibrio* community compositions and correlation of environmental factors. **(A)** The community compositions of *Vibrio* spp. along the depth at each site. **(B)** Mantel test analysis based on OTU level. ^*^P = 0.01–0.05; ^**^P = 0.001–0.01; ^***^P < 0.001. **(C)** The relationship between the top 45 dominant species and environmental factors.

**Figure 5:**
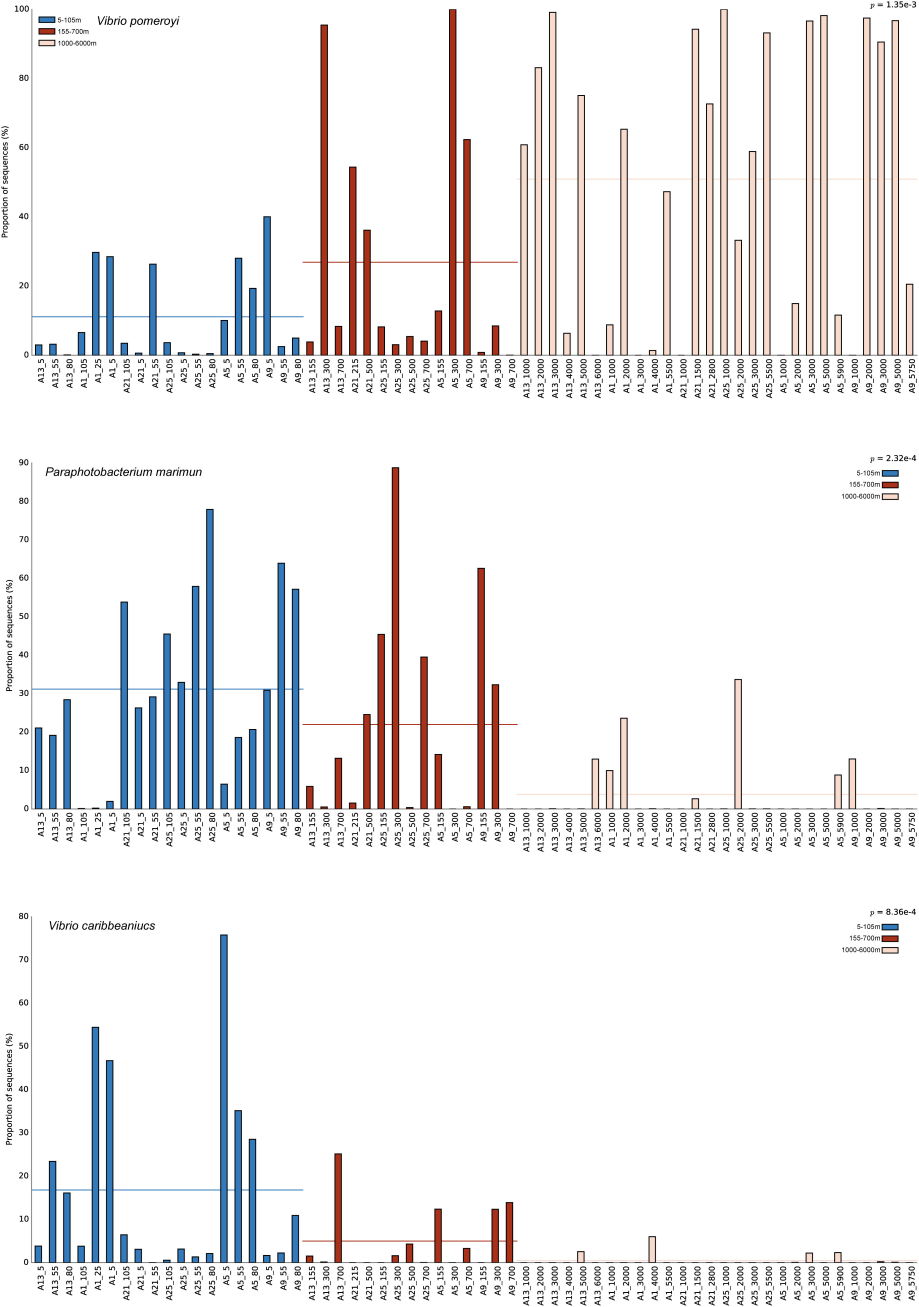
The relative abundance of *Vibrio pomeroyi, Paraphotobacterium marimun*, and *Vibrio caribbeaniucs* at different depth.

To identify specific taxa that contributed to the observed vertical dynamics of *Vibrio* communities, representative sequences of each OTU were compared against the EzBioCloud database to do the accurate identification. Almost all sequences (96.17%) belonged to the *Vibrionaceae* family, and 66.25% were assigned to the genus *Vibrio*. Forty-five abundant species (relative abundance >0.1%) were found in total samples across all sites, and accounted for 97.14% of all sequences (Figure 4A). *V. pomeryi* occupied the highest relative abundance across all samples (32.45%), followed by *V. sagamiensis* (16.42%), *P. marinum* (16.27%), *P. phosphoreum* (6.95%) and *V. caribbeanicus* (6.50%; Figure 4A). *V. pomeroyi, P. marinum*, and *V. caribbeanicus* were detected along the whole water column, whereas their relative abundances significantly varied among the different depth groups (Tukey, *P* < 0.001, Figure 5). *V. sagamiensis* and *P. marinum* were the dominant species in the UL, *V. caribbeanicus, V. campbellii* and *P. marinum* were concentrated in the UL and ML, and *V. pomeroyi* exhibited a high relative abundance in the BL (Figure 4A). In addition, different from other sites, *V. hangzhouensis* and *Vibrio* sp. OTU380 showed high relative abundance in the UL at station P1-19-1.

### The effects of environmental factors on *Vibrio* community

Distance-based redundancy analysis (db-RDA) was performed to assess the impacts of environmental parameters on the composition of *Vibrio* communities. Temperature, ${\text{SiO}}_{3}^{2-}$, ${\text{NO}}_{3}^{-}$ and ${\text{PO}}_{4}^{3-}$ drove the composition of *Vibrio* communities (Figure 3D). Further, the Mantel test showed that temperature, salinity, DO, ${\text{NO}}_{3}^{-}$, ${\text{PO}}_{4}^{3-}$ and ${\text{SiO}}_{3}^{2-}$ have significant effects on shaping the community diversities of vibrios across the vertical profile (*P* < 0.05, Figure 4B). The correlation between the relative abundance of *Vibrio* (>0.1%) and environmental parameters was calculated by Spearman's rank correlation coefficients (Figure 4C). Most of the abundant species showed significant positive correlations with temperature and DO, whereas significantly negatively correlated with depth and concentrations of ${\text{NO}}_{3}^{-}$, ${\text{PO}}_{4}^{3-}$ and ${\text{SiO}}_{3}^{2-}$ (Figure 4C). In detail, *V. pomeryi*, the most abundant species, had significant correlations with ${\text{SiO}}_{3}^{2-}$ (*P* < 0.05). *V. caribbeanicus, V. campbellii*, and *P. marinum*, the dominant species in the UL and ML, were positively related to temperature and DO, and negatively to depth, ${\text{PO}}_{4}^{3-}$, ${\text{SiO}}_{3}^{2-}$ and ${\text{NO}}_{3}^{-}$ (*P* < 0.05); whereas *Photobacterium phosphoreum*, the dominant species in the BL, positively correlated to temperature, and negatively to depth and ${\text{SiO}}_{3}^{2-}$ (*P* < 0.05). The abundant species in the UL at site P1-19-1, *Vibrio* sp. OTU380 showed negative correlations with depth and salinity, and positive correlations with DO (*P* < 0.05; Figure 4C).

### Community assembly process of *Vibrio* spp.

**Figure 6:**
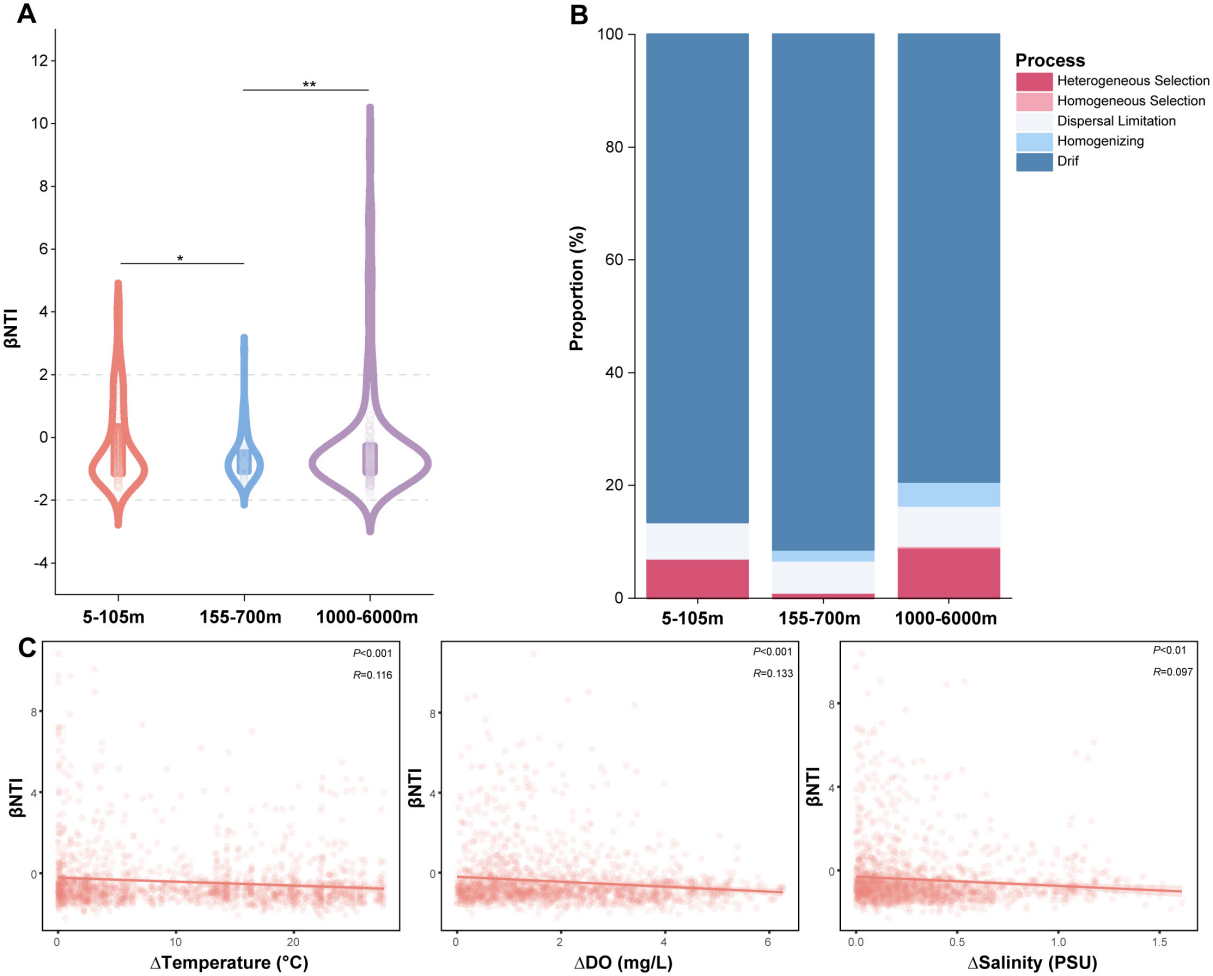
Environmental heterogeneity driving *Vibrio* community assemblage mechanisms along a vertical dimension. **(A)** The βNTI of *Vibrio* community at 5-105 m, 155-700 m, and 1000-6000 m. ^*^P < 0.05; ^**^P < 0.01. **(B)** The patterns of *Vibrio* community assembly processes at 5-105 m, 155-700 m, and 1000-6000 m. **(C)** Relationships between βNTI and differences in temperature, DO and salinity.

We used the βNTI metric to quantify the relative importance of deterministic (|βNTI|>2) or stochastic (|βNTI| < 2) factors to community structure. The results revealed that the value of βNTI were mainly distributed between −2 and 2, indicating that stochastic processes dominated the formation of *Vibrio* communities (Figure 6A). And, there was a significant difference in βNTI between the UL and ML (Kruskal-wallis, *P* < 0.05), the ML and BL (Kruskal-wallis, *P* < 0.01; Figure 6A). Meanwhile, the null model was used to explore the compositional processes of *Vibrio* communities among the UL, ML, and BL, including deterministic processes (i.e., heterogeneous and homogeneous selection) and stochastic processes (i.e., homogenizing dispersal, ecological drift and dispersal limitation). Throughout the water column, drift was the most significant process, governing the process of community assembly (Figure 6B). Heterogeneous selection had a greater impact on the UL and BL compared to the ML (Figure 6B). The relationships between βNTI and differences in temperature, DO and salinity were further analyzed, and the significant correlations were found (*P* < 0.01; Figure 6C). Increases in temperature, DO and salinity led to the elevated stochasticity of *Vibrio* community assembly, weakening environmental selection (Figure 6C).

## Discussion

*Vibrio* spp. exhibit remarkable adaptability to diverse environmental conditions, particularly in the marginal seas (Liang et al., 2019; Wang et al., 2020b; Zhu et al., 2023). However, their ecological distribution and significance of vibrios in the open ocean remain poorly understood. The NPO features great depths and highly dynamic vertical stratification, and investigating *Vibrio* dynamics along broad depth gradients can provide valuable insights. In this study, we examined the vertical distribution pattern of *Vibrio* spp. in the water column of the NPO and identified distinct community structures across different layers. Significant differences in abundance and species composition were observed among the UL, ML, and BL, reflecting the influence of environmental factors and ecological processes. Our findings help enhance the knowledge on the distribution patterns of vibrios in the open oceans, leading to offer new perspectives on their depth-related variability.

### The different *Vibrio* abundance among sites may relate to local conditions

qPCR analysis for *Vibrio* spp. abundance across different depths and locations has provided valuable insights into the intricate connections between microbial distribution and environmental gradients (Diner et al., 2021). In the vertical distribution of marine microbes, most studies have reported a general decrease in abundance with depth (Treusch et al., 2009). In this study, without any surprise, significantly higher abundances were observed in the UL with the values ranging from 5.66 × 10^4^ to 4.53 × 10^6^ copies/L, and showed a decreasing trend to the bottom in sites P1-19-1, P1-19-21 and P1-19-25. Vertical declines in temperature, salinity, and Dissolved Oxygen (DO) are key factors shaping microbial community composition in the ocean (Brown et al., 2009; Eloe et al., 2011; Siboni et al., 2016; Zhu et al., 2023). As previously reported (Zhu et al., 2023) and shown in Supplementary Table 1, surface waters are characterized by warmer temperatures, moderate salinity, and higher oxygen levels, all of which decrease with depth. Certainly, *Vibrio* spp. distribution is also shaped by seasonal and interannual changes (Asplund et al., 2011; Liang et al., 2019), and our study is based on a single-time-point sampling which limits a comprehensive analysis of temporal dynamics and highlights the need for further investigation in the future.

*Vibrio* abundance varies across sampling sites due to unique environmental conditions. In stations P1-19-5, P1-19-9 and P1-19-13, the values below 155m were much higher than those in surface seawater (Figure 2). This phenomenon has been reported in the EITO, where the *Vibrio* abundance obviously reduced with the increasing depth of water until 2,000 m and slightly raised from 2,000m to the bottom (Zhu et al., 2023). Specific environments like water mass characteristics, biological interactions, lower temperatures, higher hydrostatic pressure, and reduced predation may increase the proliferation of vibrios (Thompson and Polz, 2006; Takemura et al., 2014; Gregg et al., 2018; Sampaio et al., 2022). Indeed, the inorganic nutrients like ${\text{NO}}_{3}^{-}$, ${\text{PO}}_{4}^{3-}$ and ${\text{SiO}}_{3}^{3-}$ showed accumulated trend from UL to BL in the NPO (Supplementary Table 1). Additionally, the growth of *Vibrio* is also positively correlated with high concentrations of organic carbon and total suspended solids (Wong et al., 2019), which may contribute to observed fluctuations (Comeau and Suttle, 2007). Due to the limitations of cruise timing and data sharing, we were unable to obtain data such as Dissolved Organic Carbon (DOC) and Particulate Organic Carbon (POC), and thus could not conduct directly correlated analyses. In future studies, more organic factors would be detected to find the main influence parameters of vibrios.

### Environmental factors and stochastic processes govern the *Vibrio* community structure

Different environmental conditions give rise to diverse microbial assemblages, and the dominant species usually show regional distribution characteristics (Wong et al., 2019). A diverse community of *Vibrio* species has been recorded in the marginal seas worldwide. In the Sydney Harbor estuary, *Vibrio* sp. OTU13800 and *V. mimicus* are the dominant groups (Siboni et al., 2016), and *V. fluvialis* in the Maowei Sea (Liang et al., 2019; Chen et al., 2020; Wang et al., 2020b, 2022). In the Indian Ocean, *P. marinum* and *V. rotiferianus* are the most abundant species (Zhu et al., 2023). Differently, *V. pomeryi, V. sagamiensis, P. marinum* and *P. phosphoreum* become the dominant species in the NPO (Figure 4A), likely due to the local environmental conditions which were selected for specific species to survive (Zhang et al., 2018; Wang et al., 2019). It has been reported that *V. pomeroyi* can utilize a lot of recalcitrant organic matters like cellobiose, and it even can grow at 4 °C (Thompson et al., 2003). *P. marinum* has been considered as specific bacteria to the pelagic environment and has existed from the surface to the deep extreme hydrothermal regions (Huang et al., 2016), and *Photobacterium* species can produce polyunsaturated fatty acid, cold-adapted lipase, esterase, and antimicrobial compounds (Nogi et al., 1998; Moi et al., 2017) to survive in deep seawater and sediment. Furthermore, *V. caribbeanicus* exhibited high relative abundance in both the ETIO and NPO, which may be attributed to its broad environmental tolerance (Liang et al., 2019; Zhu et al., 2023). It is worth noting that seasonal and interannual sampling would give more reliable results.

*Vibrio* communities are primarily structured through vertical stratification of environmental factors (Thompson and Polz, 2006; Li et al., 2020a; Zhu et al., 2023). In this study, a distinct depth-dependent stratification of *Vibrio* communities was found in the NPO (Figure 4A). While environmental variables including temperature, ${\text{SiO}}_{3}^{2-}$, ${\text{NO}}_{3}^{-}$ and ${\text{PO}}_{4}^{3-}$ contributed to shaping *Vibrio* communities (Figure 3D), stochastic processes (drift and heterogeneous selection) exerted an even greater influence on their overall assembly (Figure 6A). Drift suggests that chance significantly influences species frequencies, particularly in dynamic environments (Martiny et al., 2006), whereas heterogeneous selection may reflect more distinct environmental gradients or greater habitat heterogeneity in these zones (She et al., 2021). Among all species, *V. pomeroyi* showed the highest abundance in the BL. It can thrive in colder and deeper waters, which is more common in lower depths below 200m, and especially 1,000m (Martin-Cuadrado et al., 2007; Sutton et al., 2008; Beleneva and Kukhlevskii, 2010). The reason might be that *V. pomeroyi* positively correlated with ${\text{SiO}}_{3}^{2-}$ concentration (Figure 1B, Figure 4C), and it could thrive in deeper layers where was the silicate rich environments. In contrast, *V. sagamiensis* and *P. marinum* persist in the UL and ML with varying abundances. *P. marinum* and *V. sagamiensis* are found in various varieties of marine habitats (Wang et al., 2022; Zhao et al., 2023). For example, *P. marinum* can adapt its physiology and metabolism to cope with the changing conditions encountered across different depths (Zhu et al., 2023). In this study, *P. marinum* showed positive correlations with temperature and DO, and negatively correlated to ${\text{PO}}_{4}^{3-}$, ${\text{SiO}}_{3}^{2-}$ and ${\text{NO}}_{3}^{-}$ (Fig. 4C). Additionally, *V. hangzhouensis* and *Vibrio* sp. OTU380 showed high relative abundance in the UL at site P1-19-1. The reason may be that *Vibrio* sp. OTU380 negatively correlated to depth and salinity (Figure 4C), and *V. hangzhouensis* exhibits adaptation to cold environments where the temperature at site P1-19-1 were significantly lower than those of other sites (Figure 1B) (Xu et al., 2009).

### Nutrients from water masses and sediments may enhance the alpha diversity of *Vibrio* spp.

In this study, Shannon, Simpson and Pielou's indices increased near the bottom layers (Supplementary Figure 1), and showed positive correlations with ${\text{PO}}_{4}^{3-}$, ${\text{SiO}}_{3}^{2-}$ and ${\text{NO}}_{3}^{-}$ (Figure 3A). The vertical motions accompanied from the frontal waves along the complex water masses transferred nutrients from BL to ML (Kouketsu et al., 2007). In the study area, the Kuroshio and the Kuroshio Extension transported warm and saline water, whereas the Oyashio transported cold and low-salinity water mass with low potential vorticity characteristics (Hiroe et al., 2002; Masujima et al., 2003). Due to the different potential temperature and salinity meet along the Kuroshio Extension, there is formed a strong front of water properties (Hiroe et al., 2002; Masujima et al., 2003). These frontal waves may accompany vertical motions, for example, the upwelling associated with frontal waves in the Gulf Stream lifts nutrient-rich subsurface water to shallower depths and enhances biological productivity (Kouketsu et al., 2007). Meanwhile, nutrient enrichment may increase microbial diversity by promoting niche variations within the *Vibrio* population (Zhao et al., 2023). Depends on the physico-chemical conditions at the sediment-water interface, the resuspension may induce the benthic dynamics of inorganic nutrients, e.g., Dissolved Inorganic Phosphorus (DIP) and Dissolved Silicate (DSi) (Couceiro et al., 2013; Niemistö and Lund-Hansen, 2019). Interestingly, there are significant positive relationships between *Vibrio* diversity and ${\text{SiO}}_{3}^{2-}$. Silicate is not readily bioavailable to most organisms, and usually plays a crucial role in the growth of diatom (Wear et al., 2015). The observed correlation may reflect an indirect effect of silicate (Figure 3A), where higher silicate supports diatom growth, leading to conditions that favor a more diverse *Vibrio* community (Wear et al., 2015). However, the mechanisms for such interactions are not well-established (Von Moos and Slaveykova, 2014), and should be taken into consideration in future.

## Conclusion

Our study investigated the vertical distribution pattern of *Vibrio* communities in the NPO, revealing complex effects by environmental factors and community assembly processes. The results highlight that *Vibrio* spp. abundance and diversities varied significantly across different depths and stations, and increased near the bottom layers which may due to the nutrients transferred by vertical motions along complex water masses in the NPO. The *Vibrio* community exhibits significant stratification by depth, and specific *Vibrio* species exhibited in the distinct depth. *V. sagamiensis* and *P. marinum* dominated in the UL, whereas *V. pomeroyi* was more abundant in deeper waters. Both environmental factors (e.g., temperature and nutrient levels ${\text{NO}}_{3}^{-}$, ${\text{PO}}_{4}^{3-}$ and ${\text{SiO}}_{3}^{2-}$) and stochastic processes affected *Vibrio* community, with deterministic selection had a stronger impact in the UL and BL. Our study highlighted the complexity of *Vibrio* community distribution in the NPO, providing insights into the potential influences of complex water masses on microbial diversity and leading to the future research on their response to environmental changes.

## Data Availability

The original contributions presented in the study are publicly available. This data can be found here: https://www.ncbi.nlm.nih.gov/ accession number PRJNA1276698.

## References

[B1] Asplund M. E. Rehnstam-Holm A. S. Atnur V. Raghunath P. Saravanan V. Härnström K.. (2011). Water column dynamics of *Vibrio* in relation to phytoplankton community composition and environmental conditions in a tropical coastal area. Environ. Microbiol. 13, 2738–2751. 10.1111/j.1462-2920.2011.02545.x 21895909

[B2] Austin B. (1988). Marine Microbiology. Cambridge: Cambridge University Press.

[B3] Baffone W. Tarsi R. Pane L. Campana R. Repetto B. Mariottini G. L.. (2006). Detection of free-living and plankton-bound vibrios in coastal waters of the Adriatic Sea (Italy) and study of their pathogenicity-associated properties. Environ. Microbiol. 8, 1299–1305. 10.1111/j.1462-2920.2006.01011.x 16817938

[B4] Baker-Austin C. Oliver J. D. Alam M. Ali A. Waldor M. K. Qadri F.. (2018). *Vibrio* spp. infections. Nat. Rev. Dis. Primers 4, 1–19. 10.1038/s41572-018-0005-8 30002421

[B5] Beleneva I. Kukhlevskii A. (2010). Characterization of *Vibrio gigantis* and *Vibrio pomeroyi* isolated from invertebrates of Peter the Great Bay, Sea of Japan. Microbiology 79, 402–407. 10.1134/S0026261710030173

[B6] Brown M. V. Philip G. K. Bunge J. A. Smith M. C. Bissett A. Lauro F. M.. (2009). Microbial community structure in the North Pacific ocean. ISME J. 3, 1374–1386. 10.1038/ismej.2009.86 19626056

[B7] Brown S. A. Balmonte J. P. Hoarfrost A. Ghobrial S. Arnosti C. (2022). Depth-related patterns in microbial community responses to complex organic matter in the western North Atlantic Ocean. Biogeosciences 19, 5617–5631. 10.5194/bg-19-5617-2022

[B8] Caporaso J. G. Kuczynski J. Stombaugh J. Bittinger K. Bushman F. D. Costello E. K.. (2010). QIIME allows analysis of high-throughput community sequencing data. Nat. Methods 7, 335–336. 10.1038/nmeth.f.303 20383131 PMC3156573

[B9] Chen X. Zhao H. Jiang G. Tang J. Xu Q. Huang L.. (2020). Responses of free-living vibrio community to seasonal environmental variation in a Subtropical Inland Bay. Front. Microbiol. 11:610974. 10.3389/fmicb.2020.610974 33381102 PMC7767907

[B10] Comeau A. M. Suttle C. A. (2007). Distribution, genetic richness and phage sensitivity of *Vibrio* spp. from coastal British Columbia. Environ. Microbiol. 9, 1790–1800. 10.1111/j.1462-2920.2007.01299.x 17564612

[B11] Corzett C. H. Elsherbini J. Chien D. M. Hehemann J.-H. Henschel A. Preheim S. P.. (2018). Evolution of a vegetarian *vibrio*: metabolic specialization of *Vibrio breoganii* to macroalgal substrates. J. Bacteriol. 200:e00020-18. 10.1128/JB.00020-18 29632094 PMC6040190

[B12] Couceiro F. Fones G. R. Thompson C. E. Statham P. J. Sivyer D. B. Parker R.. (2013). Impact of resuspension of cohesive sediments at the Oyster Grounds (North Sea) on nutrient exchange across the sediment–water interface. Biogeochemistry 113, 37–52. 10.1007/s10533-012-9710-7

[B13] Dahm C. N. Grimm N. B. Marmonier P. Valett H. M. Vervier P. (1998). Nutrient dynamics at the interface between surface waters and groundwaters. Freshw. Biol. 40, 427–451. 10.1046/j.1365-2427.1998.00367.x

[B14] De Graaf F. Ford H. L. Burls N. Brown R. Brierley C. Foster G. L.. (2025). Reduced North Pacific Deep Water formation across the Northern Hemisphere glaciation. Nat. Commun. 16:2704. 10.1038/s41467-025-58069-x 40108166 PMC11923177

[B15] Deng J.-J. Zhang J.-R. Mao H.-H. Zhang M.-S. Lu Y.-S. Luo X.-C. (2025). Chitinases are important virulence factors in Vibrio for degrading the chitin-rich barrier of shrimp. Int. J. Biol. Macromol. 293:139215. 10.1016/j.ijbiomac.2024.139215 39732246

[B16] Diner R. E. Kaul D. Rabines A. Zheng H. Steele J. A. Griffith J. F.. (2021). Pathogenic *Vibrio* species are associated with distinct environmental niches and planktonic taxa in Southern California (USA) aquatic microbiomes. Msystems 6:e0057121. 10.1128/msystems.00571-21 34227831 PMC8407410

[B17] Doni L. (2024). Global Biogeography and Ecology of Vibrio in a Warming Planet. Genoa: University of Genoa Press.

[B18] Edgar R. C. Haas B. J. Clemente J. C. Quince C. Knight R. (2011). UCHIME improves sensitivity and speed of chimera detection. Bioinformatics 27, 2194–2200. 10.1093/bioinformatics/btr381 21700674 PMC3150044

[B19] Eloe E. A. Shulse C. N. Fadrosh D. W. Williamson S. J. Allen E. E. Bartlett D. H. (2011). Compositional differences in particle-associated and free-living microbial assemblages from an extreme deep-ocean environment. Environ. Microbiol. Rep. 3, 449–458. 10.1111/j.1758-2229.2010.00223.x 23761307

[B20] Fenies P. Bassetti M.-A. Riveiros N. V. Menniti C. Frigola C. Babonneau N.. (2023). Changes in Kuroshio current dynamics and East Asian monsoon variability during the last 26 kyr. Palaeogeogr. Palaeoclimatol. Palaeoecol. 632:111836. 10.1016/j.palaeo.2023.111836

[B21] Grasshoff K. Kremling K. Ehrhardt M. (2009). Methods of Seawater Analysis. New York, NY: John Wiley & Sons Press.

[B22] Gregg M. C. D'Asaro E. A. Riley J. J. Kunze E. (2018). Mixing efficiency in the ocean. Ann. Rev. Mar. Sci. 10, 443–473. 10.1146/annurev-marine-121916-063643 28934598

[B23] Gyraite G. Katarzyte M. Schernewski G. (2019). First findings of potentially human pathogenic bacteria Vibrio in the south-eastern Baltic Sea coastal and transitional bathing waters. Mar. Pollut. Bull. 149:110546. 10.1016/j.marpolbul.2019.110546 31543486

[B24] Hiroe Y. Yasuda I. Komatsu K. Kawasaki K. Joyce T. M. Bahr F. (2002). Transport of North Pacific intermediate water in the Kuroshio–Oyashio interfrontal zone. Deep Sea Res. 2 Top. Stud. Oceanogr. 49, 5353–5364. 10.1016/S0967-0645(02)00195-9

[B25] Hu Y. Shao W. Shen W. Zuo J. Jiang T. Hu S. (2024). Analysis of sea surface temperature cooling in typhoon events passing the Kuroshio current. J. Ocean Univ. China 23, 287–303. 10.1007/s11802-024-5608-y

[B26] Huang Z. Dong C. Shao Z. (2016). Paraphotobacterium marinum gen. nov., sp. nov., a member of the family *Vibrionaceae*, isolated from surface seawater. Int. J. Syst. Evol. Microbiol. 66, 3050–3056. 10.1099/ijsem.0.001142 27154455

[B27] Hutchins D. A. Fu F. (2017). Microorganisms and ocean global change. Nat. Microbiol. 2, 1–11. 10.1038/nmicrobiol.2017.58 28540925

[B28] Jesser K. Noble R. (2018). Characterizing the ecology of *Vibrio* in the Neuse River Estuary, North Carolina using heat shock protein 60 (hsp60) next-generation amplicon sequencing. Appl. Environ. Microbiol. 84, e00333–e00318. 10.1128/AEM.00333-18 29678912 PMC6007114

[B29] Johnson C. N. (2015). Influence of environmental factors on *Vibrio* spp. in coastal ecosystems. Microbiol. Spectr. 3, VE–0008-2014. 10.1128/microbiolspec.ve-0008-2014 26185069

[B30] Johnson G. C. Lyman J. M. (2022). GOSML: a global ocean surface mixed layer statistical monthly climatology: means, percentiles, skewness, and kurtosis. J. Geophys. Res. Oceans 127:e2021JC018219. 10.1029/2021JC018219

[B31] Kopprio G. A. Streitenberger M. E. Okuno K. Baldini M. Biancalana F. Fricke A.. (2017). Biogeochemical and hydrological drivers of the dynamics of *Vibrio* species in two Patagonian estuaries. Sci. Total Environ. 579, 646–656. 10.1016/j.scitotenv.2016.11.045 27871750

[B32] Kouketsu S. Yasuda I. Hiroe Y. (2007). Three-dimensional structure of frontal waves and associated salinity minimum formation along the Kuroshio Extension. J. Phys. Oceanogr. 37, 644–656. 10.1175/JPO3026.1

[B33] Lauro F. M. McDougald D. Thomas T. Williams T. J. Egan S. Rice S.. (2009). The genomic basis of trophic strategy in marine bacteria. Proceed. Nat. Acad. Sci. 106, 15527–15533. 10.1073/pnas.0903507106 19805210 PMC2739866

[B34] Li B. Liu J. Zhou S. Fu L. Yao P. Chen L.. (2020a). Vertical variation in Vibrio community composition in Sansha Yongle Blue Hole and its ability to degrade macromolecules. Mar. Life Sci. Technol. 2, 60–72. 10.1007/s42995-019-00003-4

[B35] Li N. Dong K. Jiang G. Tang J. Xu Q. Li X.. (2020b). Stochastic processes dominate marine free-living *Vibrio* community assembly in a subtropical gulf. FEMS Microbiol. Ecol. 96:fiaa198. 10.1093/femsec/fiaa198 32990746

[B36] Liang J. Liu J. Wang X. Lin H. Liu J. Zhou S.. (2019). Spatiotemporal dynamics of free-living and particle-associated *Vibrio* communities in the northern Chinese marginal seas. Appl. Environ. Microbiol. 85, e00217–00219. 10.1128/AEM.00217-19 30824453 PMC6495765

[B37] Liu Y. Qiu Y. Li D. Artemova A. V. Zhang Y. Bosin A. A.. (2022). Abrupt fluctuations in North Pacific Intermediate Water modulated changes in deglacial atmospheric CO2. Front. Mar. Sci. 9, 945110. 10.3389/fmars.2022.945110

[B38] Martin-Cuadrado A.-B. Lopez-Garcia P. Alba J.-C. Moreira D. Monticelli L. Strittmatter A.. (2007). Metagenomics of the deep Mediterranean, a warm bathypelagic habitat. PLoS ONE 2:e914. 10.1371/journal.pone.0000914 17878949 PMC1976395

[B39] Martinez-Urtaza J. Blanco-Abad V. Rodriguez-Castro A. Ansede-Bermejo J. Miranda A. Rodriguez-Alvarez M. X. (2012). Ecological determinants of the occurrence and dynamics of Vibrio parahaemolyticus in offshore areas. ISME J. 6, 994–1006. 10.1038/ismej.2011.156 22094349 PMC3329108

[B40] Martiny J. B. H. Bohannan B. J. Brown J. H. Colwell R. K. Fuhrman J. A. Green J. L.. (2006). Microbial biogeography: putting microorganisms on the map. Nat. Rev. Microbiol. 4, 102–112. 10.1038/nrmicro1341 16415926

[B41] Masujima M. Yasuda I. Hiroe Y. Watanabe T. (2003). Transport of Oyashio water across the subarctic front into the mixed water region and formation of NPIW. J. Oceanogr. 59, 855–869. 10.1023/B:JOCE.0000009576.09079.f5

[B42] Maximenko N. A. Shcherbina A. Y. (1996). “Fine-structure of the North Pacific Intermediate Water Layer”, in: *Report of The Pices Workshop on The Okhotsk Sea and Adjacent Areas Outline of the Workshop 2. Summary reports from sessions* 3. Recommendations of the workshop 10, 104.

[B43] Moi I. M. Roslan N. N. Leow A. T. C. Ali M. S. M. Rahman R. N. Z. R.A. Rahimpour A.. (2017). The biology and the importance of Photobacterium species. Appl. Microbiol. Biotechnol. 101, 4371–4385. 10.1007/s00253-017-8300-y 28497204

[B44] Naylor D. McClure R. Jansson J. (2022). Trends in microbial community composition and function by soil depth. Microorganisms 10, 540. 10.3390/microorganisms10030540 35336115 PMC8954175

[B45] Niemistö J. Lund-Hansen L. C. (2019). Instantaneous effects of sediment resuspension on inorganic and organic benthic nutrient fluxes at a shallow water coastal site in the Gulf of Finland, Baltic Sea. ESCO 42, 2054–2071. 10.1007/s12237-019-00648-5

[B46] Nogi Y. Masui N. Kato C. (1998). Photobacterium profundum sp. nov., a new, moderately barophilic bacterial species isolated from a deep-sea sediment. Extremophiles 2, 1–8. 10.1007/s007920050036 9676237

[B47] Onohuean H. Agwu E. Nwodo U. (2022). A global perspective of *Vibrio* species and associated diseases: three-decade meta-synthesis of research advancement. Environ. Health Insights 16:11786302221099406. 10.1177/11786302221099406 35601189 PMC9121474

[B48] Parks D. H. Tyson G. W. Hugenholtz P. Beiko R. G. (2014). STAMP: statistical analysis of taxonomic and functional profiles. Bioinformatics 30, 3123–3124. 10.1093/bioinformatics/btu494 25061070 PMC4609014

[B49] Parsons T. R Maita Y Lalli C. M. (1984). “Determination of chlorophylls and total carotenoids: spectrophotometric method,” in A Manual of Chemical & Biological Methods for Seawater Analysis, eds. T. R. Parsons, Y. Maita, and C. M. Lalli (Amsterdam: Pergamon), 101–104. 10.1016/b978-0-08-030287-4.50032-3

[B50] Qiu B. (2001). “Kuroshio and Oyashio currents”, in *Encyclopedia of Ocean Sciences* (Cambridge: Academic Press), 1413–1425.

[B51] Roch M. Brandt P. Schmidtko S. (2023). Recent large-scale mixed layer and vertical stratification maxima changes. Front. Mar. Sci. 10:1277316. 10.3389/fmars.2023.1277316

[B52] Rogers A. D. (2015). Environmental change in the deep ocean. Ann. Rev. Environ. Res. 40, 1–38. 10.1146/annurev-environ-102014-021415

[B53] Sampaio A. Silva V. Poeta P. Aonofriesei F. (2022). *Vibrio* spp.: life strategies, ecology, and risks in a changing environment. Diversity 14:97. 10.3390/d14020097

[B54] Sérazin G. Tréguier A. M. de Boyer Montégut C. (2023). A seasonal climatology of the upper ocean pycnocline. Front. Mar. Sci. 10:1120112. 10.3389/fmars.2023.1120112

[B55] She Z. Pan X. Wang J. Shao R. Wang G. Wang S.. (2021). Vertical environmental gradient drives prokaryotic microbial community assembly and species coexistence in a stratified acid mine drainage lake. Water Res. 206:117739. 10.1016/j.watres.2021.117739 34653798

[B56] Shi Y. Li Y. Xiang X. Sun R. Yang T. He D.. (2018). Spatial scale affects the relative role of stochasticity versus determinism in soil bacterial communities in wheat fields across the North China Plain. Microbiome 6:27. 10.1186/s40168-018-0409-4 29402331 PMC5799910

[B57] Siboni N. Balaraju V. Carney R. Labbate M. Seymour J. R. (2016). Spatiotemporal dynamics of *Vibrio* spp. within the Sydney Harbour estuary. Front. Microbiol. 7:460. 10.3389/fmicb.2016.00460 27148171 PMC4829023

[B58] Stegen J. C. Lin X. Fredrickson J. K. Chen X. Kennedy D. W. Murray C. J.. (2013). Quantifying community assembly processes and identifying features that impose them. ISME J. 7, 2069–2079. 10.1038/ismej.2013.93 23739053 PMC3806266

[B59] Sutton T. Porteiro F. Heino M. Byrkjedal I. Langhelle G. Anderson C.. (2008). Vertical structure, biomass and topographic association of deep-pelagic fishes in relation to a mid-ocean ridge system. Deep Sea Res. 2 Top. Stud. Oceanogr. 55, 161–184. 10.1016/j.dsr2.2007.09.013

[B60] Takemura A. F. Chien D. M. Polz M. F. (2014). Associations and dynamics of Vibrionaceae in the environment, from the genus to the population level. Front. Microbiol. 5:38. 10.3389/fmicb.2014.00038 24575082 PMC3920100

[B61] Thompson F. Thompson C. Li Y. Gomez-Gil B. Vandenberghe J. Hoste B.. (2003). Vibrio kanaloae sp. nov., *Vibrio pomeroyi* sp. nov. and *Vibrio chagasii* sp. nov., from sea water and marine animals. Int. J. Syst. Evol. Microbiol. 53, 753–759. 10.1099/ijs.0.02490-0 12807197

[B62] Thompson F. L. Iida T. Swings J. (2004a). Biodiversity of vibrios. Microbiol. Mol. Biol. Rev. 68, 403–431. 10.1128/MMBR.68.3.403-431.2004 15353563 PMC515257

[B63] Thompson J. R. Polz M. F. (2006). “Dynamics of Vibrio populations and their role in environmental nutrient cycling,” in The Biology of Vibrios, eds F. L. Thompson, B. Austin, and J. Swings (Washington, DC: ASM Press), 190–203.

[B64] Thompson J. R. Randa M. A. Marcelino L. A. Tomita-Mitchell A. Lim E. Polz M. F. (2004b). Diversity and dynamics of a North Atlantic coastal Vibrio community. Appl. Environ. Microbiol. 70, 4103–4110. 10.1128/AEM.70.7.4103-4110.2004 15240289 PMC444776

[B65] Treusch A. H. Vergin K. L. Finlay L. A. Donatz M. G. Burton R. M. Carlson C. A.. (2009). Seasonality and vertical structure of microbial communities in an ocean gyre. ISME J. 3, 1148–1163. 10.1038/ismej.2009.60 19494846

[B66] Vezzulli L. Pezzati E. Brettar I. Hofle M. Pruzzo C. (2015). Effects of global warming on Vibrio ecology. Microbiol. Spectr. 3, VE-0004-2014. 10.1128/microbiolspec.VE-0004-2014 26185070

[B67] Von Moos N. Slaveykova V. I. (2014). Oxidative stress induced by inorganic nanoparticles in bacteria and aquatic microalgae–state of the art and knowledge gaps. Nanotoxicology 8, 605–630. 10.3109/17435390.2013.809810 23738945

[B68] Wang H. N. Du G. X. Yu S. X. Zhang H. H. Song G. D. Liu S. M.. (2024). Methane distribution, production, and emission in the Western North Pacific. J. Geophys. Res. Oceans 129:e2023JC020482. 10.1029/2023JC02048237922593

[B69] Wang M. Ma Y. Feng C. Cai L. Li W. (2020a). Diversity of pelagic and benthic bacterial assemblages in the western Pacific Ocean. Front. Microbiol. 11:1730. 10.3389/fmicb.2020.01730 33071990 PMC7533643

[B70] Wang Q. Garrity G. M. Tiedje J. M. Cole J. R. (2007). Naive Bayesian classifier for rapid assignment of rRNA sequences into the new bacterial taxonomy. Appl. Environ. Microbiol. 73, 5261–5267. 10.1128/AEM.00062-07 17586664 PMC1950982

[B71] Wang X. Liu J. Li B. Liang J. Sun H. Zhou S.. (2019). Spatial heterogeneity of *Vibrio* spp. in sediments of Chinese marginal seas. Appl. Environ. Microbiol. 85, e03064–e03018. 10.1128/AEM.03064-18 30877118 PMC6498182

[B72] Wang X. Liu J. Liang J. Sun H. Zhang X. H. (2020b). Spatiotemporal dynamics of the total and active *Vibrio* spp. populations throughout the Changjiang estuary in China. Environ. Microbiol. 22, 4438–4455. 10.1111/1462-2920.15152 33462948 PMC7689709

[B73] Wang X. Liu J. Zhao W. Liu J. Liang J. Thompson F.. (2022). Fine-scale structuring of planktonic *Vibrio* spp. in the Chinese marginal seas. Appl. Environ. Microbiol. 88, e01262–e01222. 10.1128/aem.01262-22 36346224 PMC9746320

[B74] Wear E. K. Carlson C. A. Windecker L. A. Brzezinski M. A. (2015). Roles of diatom nutrient stress and species identity in determining the short-and long-term bioavailability of diatom exudates to bacterioplankton. Mar. Chem. 177, 335–348. 10.1016/j.marchem.2015.09.001

[B75] Westrich J. R. Ebling A. M. Landing W. M. Joyner J. L. Kemp K. M. Griffin D. W.. (2016). Saharan dust nutrients promote *Vibrio* bloom formation in marine surface waters. Proceed. Nat. Acad. Sci. 113, 5964–5969. 10.1073/pnas.1518080113 27162369 PMC4889353

[B76] Williams N. L. Siboni N. King W. L. Balaraju V. Bramucci A. Seymour J. R. (2022). Latitudinal dynamics of *Vibrio* along the Eastern Coastline of Australia. Water 14:2510. 10.3390/w14162510

[B77] Wong Y. Y. Lee C. W. Bong C. W. Lim J. H. Narayanan K. Sim E. U. H. (2019). Environmental control of *Vibrio* spp. abundance and community structure in tropical waters. FEMS Microbiol. Ecol. 95:fiz176. 10.1093/femsec/fiz176 31688899

[B78] Xu X.-W. Wu Y.-H. Wang C.-S. Oren A. Wu M. (2009). *Vibrio hangzhouensis* sp. nov., isolated from sediment of the East China Sea. Int. J. Syst. Evol. Microbiol. 59, 2099–2103. 10.1099/ijs.0.008698-0 19605706

[B79] Yin Q. Fu B. Li B. Shi X. Inagaki F. Zhang X.-H. (2013). Spatial variations in microbial community composition in surface seawater from the ultra-oligotrophic center to rim of the South Pacific Gyre. PLoS ONE 8:e55148. 10.1371/journal.pone.0055148 23405118 PMC3566182

[B80] Yu S.-X. Wang X. Wang Y. Wang H. Liu J. Hong W.. (2025). Diverse marine *Vibrio* species convert methylphosphonate to methane. *Mar. Life Sci. Technol*. 1-15. 10.1007/s42995-025-00278-w PMC12413358 40919471

[B81] Zhang M. Wang J. Zeng R. Wang D. Wang W. Tong X.. (2022). Agarose-degrading characteristics of a deep-sea bacterium *Vibrio* natriegens WPAGA4 and its cold-adapted GH50 agarase Aga3420. Mar. Drugs 20:692. 10.3390/md20110692 36355015 PMC9698624

[B82] Zhang X. Lin H. Wang X. Austin B. (2018). Significance of *Vibrio* species in the marine organic carbon cycle—a review. Sci. China Earth Sci. 61, 1357–1368. 10.1007/s11430-017-9229-x

[B83] Zhao W. Chen X. Liu R. Tian P. Niu W. Zhang X.-H.. (2023). Distinct coral environments shape the dynamic of planktonic *Vibrio* spp. Environ. Microbiome 18:77. 10.1186/s40793-023-00532-7 37872593 PMC10594878

[B84] Zhu S. Wang X. Zhao W. Zhang Y. Song D. Cheng H.. (2023). Vertical dynamics of free-living and particle-associated vibrio communities in the eastern tropical Indian Ocean. Front. Microbiol. 14:1285670. 10.3389/fmicb.2023.1285670 37928659 PMC10620696

