# Supplementary material for: Vertical dynamic patterns of Vibrio spp. in the northwestern Pacific Ocean
Source: Front Microbiol. 2025 Sep 8;16:1649301. doi: 10.3389/fmicb.2025.1649301 (PMC12450871; doi:10.3389/fmicb.2025.1649301)

Supplementary materials for:

Vertical dynamic patterns of *Vibrio* spp. in the northwestern Pacific Ocean

Leihaothabam Jeeny[1,2,3†], Keyi Huang[1,2,3†], Xing Chen[1,2,3], Yan Wang[1,2,3], Shaodong Zhu[1,2,3], Yulin Zhang[1,2,3], Xiao-Hua Zhang[1,2,3] and Xiaolei Wang[1,2,3*]

[1]Frontiers Science Center for Deep Ocean Multispheres and Earth System, and College of Marine Life Sciences, Ocean University of China, Qingdao, China

[2]Laboratory for Marine Ecology and Environmental Science, Qingdao Marine Science and Technology Center, Qingdao, China

[3]Key Laboratory of Evolution and Marine Biodiversity (Ministry of Education), Institute of Evolution and Marine Biodiversity, Ocean University of China, Qingdao 266003, China

[*]Author for correspondence:

Xiaolei Wang, E-mail: wangxiaolei@ouc.edu.cn

[†]These authors contributed equally to this work

Running title: Vertical distribution of *Vibrio* spp. in the Pacific Ocean

Table S1. The environmental parameters and the abundance of total vibrios in each sampling sites.

Station	Depth (m)	T (°C)	Salinity (PSU)	DO (mg/L)	PO_4^{3-} (μM/L)	SiO_3^{2-} (μM/L)	NO_2^- (μM/L)	NO_3^- (μM/L)	Total *Vibrio* (copies/L)
P1-19-1	5	14.984	33.609	7.51	2.73	136.54	0.08	35.33	3.69×10^6
	25	15.041	33.622	7.44	2.55	148.38	0.04	34.55	4.53×10^6
	105	5.224	33.514	7.52	0.39	6.42	0.19	2.61	2.98×10^6
	1000	2.875	34.411	1.26	1.64	30.37	0.08	21.68	1.52×10^5
	2000	1.874	34.599	2.95	2.58	96.72	0.07	35.02	9.87×10^4
	3000	1.544	34.658	3.98	2.83	140.38	0.04	38.29	-
	4000	1.479	34.678	4.95	2.85	153.97	0.06	38.10	5.34×10^4
	5500	1.585	34.687	5.09	2.63	156.16	0.06	36.67	1.40×10^5
P1-19-5	5	23.910	34.498	6.29	0.10	3.02	0.07	N.D.	2.00×10^6
	55	23.932	34.497	6.27	0.08	1.33	0.07	N.D.	1.77×10^6
	80	23.769	34.545	5.91	0.11	1.81	0.15	0.60	2.17×10^5
	155	19.062	34.782	5.68	0.33	4.57	0.06	4.14	7.22×10^5

	**300**	**15.815**	**34.646**	**5.67**	**0.54**	**9.18**	**0.06**	**7.60**	**9.52×10^{6}**
	**700**	**4.634**	**34.152**	**2.13**	**2.53**	**95.33**	**0.04**	**34.33**	**7.34×10^{5}**
	**1000**	**3.317**	**34.350**	**1.26**	**2.82**	**130.22**	**0.04**	**37.61**	**3.39×10^{4}**
	**2000**	**1.947**	**34.590**	**2.53**	**2.73**	**162.59**	**0.05**	**37.23**	**2.31×10^{4}**
	**3000**	**1.546**	**34.659**	**4.07**	**2.51**	**156.98**	**0.05**	**34.61**	**8.37×10^{6}**
	**5000**	**1.511**	**34.688**	**5.19**	**2.32**	**144.80**	**0.05**	**32.19**	**1.25×10^{6}**
	**5900**	**1.601**	**34.692**	**5.39**	**2.30**	**138.98**	**0.05**	**32.12**	**2.43×10^{7}**
**P1-19-9**	**5**	**25.597**	**34.698**	**6.75**	**0.07**	**2.90**	**0.04**	**N.D.**	**9.99×10^{5}**
	**55**	**24.991**	**34.731**	**7.04**	**0.06**	**1.05**	**0.03**	**N.D.**	**5.20×10^{5}**
	**80**	**20.515**	**34.858**	**7.46**	**0.07**	**1.44**	**0.06**	**N.D.**	**4.58×10^{5}**
	**155**	**17.927**	**34.798**	**7.09**	**0.27**	**2.53**	**0.08**	**2.87**	**4.43×10^{5}**
	**300**	**16.754**	**34.742**	**6.84**	**0.35**	**4.64**	**0.05**	**4.62**	**1.34×10^{5}**
	**700**	**7.036**	**34.013**	**5.09**	**1.76**	**43.29**	**0.05**	**23.88**	**7.89×10^{5}**
	**1000**	**4.166**	**34.216**	**2.31**	**2.65**	**108.24**	**0.04**	**36.40**	**4.08×10^{4}**
	**2000**	**2.025**	**34.579**	**2.31**	**2.68**	**162.13**	**0.04**	**37.07**	**7.07×10^{4}**
	**3000**	**1.553**	**34.600**	**4.27**	**2.44**	**155.46**	**0.04**	**34.49**	**3.50×10^{6}**

	5000	1.516	34.688	5.54	2.31	147.45	0.04	32.38	1.76×10^{7}
	5750	1.581	34.691	5.56	2.30	137.89	0.04	31.94	2.14×10^{4}
P1-19-13	5	27.181	34.901	6.27	0.04	2.54	0.05	N.D.	6.84×10^{5}
	55	26.523	34.915	6.82	0.06	0.94	0.05	N.D.	2.21×10^{5}
	80	21.357	34.891	7.17	0.06	1.30	0.05	N.D.	1.59×10^{5}
	155	17.922	34.807	6.52	0.31	2.67	0.05	3.37	5.96×10^{6}
	300	15.871	34.659	6.11	0.49	7.32	0.05	6.98	1.77×10^{5}
	700	5.938	34.033	3.74	2.17	65.59	0.05	28.81	6.27×10^{5}
	1000	3.800	34.279	1.42	2.84	117.97	0.04	37.26	5.62×10^{4}
	2000	1.962	34.598	2.97	2.72	155.39	0.04	36.53	6.38×10^{4}
	3000	1.597	34.657	4.35	2.53	151.31	0.05	34.52	2.14×10^{6}
	4000	1.495	34.679	4.90	2.35	146.37	0.05	33.52	1.13×10^{6}
	5000	1.517	34.687	5.24	2.28	145.09	0.05	32.21	1.48×10^{6}
	6000	1.620	34.691	5.36	2.32	140.32	0.05	32.34	1.06×10^{7}
P1-19-21	5	27.516	34.856	6.35	0.07	1.07	0.05	N.D.	1.57×10^{6}
	55	21.880	35.028	6.02	0.11	1.50	0.04	0.14	1.19×10^{5}

	**105**	**19.957**	**34.944**	**5.78**	**0.26**	**2.19**	**0.04**	**2.08**	**5.66×10^4**
	**215**	**15.984**	**34.659**	**6.33**	**0.38**	**5.81**	**0.06**	**5.34**	**8.17×10^4**
	**500**	**7.881**	**34.093**	**4.42**	**1.80**	**46.96**	**0.05**	**24.60**	**4.69×10^4**
	**1000**	**3.922**	**34.482**	**2.25**	**2.70**	**114.43**	**0.03**	**36.04**	**4.02×10^4**
	**1500**	**2.560**	**34.567**	**3.01**	**2.62**	**141.00**	**0.04**	**35.11**	**1.04×10^5**
	**2800**	**1.688**	**34.652**	**4.12**	**2.49**	**154.34**	**0.03**	**33.98**	**4.28×10^4**
**P1-19-25**	**5**	**29.133**	**34.610**	**6.11**	**0.06**	**0.67**	**0.03**	**N.D.**	**1.67×10^6**
	**55**	**28.896**	**34.674**	**6.21**	**0.07**	**0.19**	**0.03**	**N.D.**	**3.92×10^5**
	**80**	**25.975**	**35.070**	**6.34**	**0.06**	**0.41**	**0.03**	**N.D.**	**6.90×10^4**
	**105**	**24.574**	**35.124**	**6.43**	**0.07**	**0.28**	**0.03**	**0.13**	**2.23×10^5**
	**155**	**21.592**	**35.024**	**6.13**	**0.13**	**1.16**	**0.08**	**0.48**	**9.93×10^4**
	**300**	**N.D.**	**N.D.**	**5.86**	**0.31**	**10.98**	**0.05**	**8.81**	**5.79×10^4**
	**500**	**7.275**	**34.285**	**2.34**	**0.70**	**57.51**	**0.03**	**29.58**	**3.96×10^4**
	**700**	**5.535**	**34.444**	**2.26**	**2.37**	**82.97**	**0.03**	**32.90**	**2.32×10^5**
	**1000**	**4.049**	**34.524**	**2.56**	**2.63**	**110.50**	**0.03**	**33.09**	**5.10×10^5**
	**2000**	**2.092**	**34.623**	**3.66**	**2.60**	**149.19**	**0.06**	**33.06**	**6.42×10^4**

**3000**	**1.606**	**34.666**	**4.61**	**2.53**	**156.37**	**0.04**	**31.58**	**1.72×10^5**
**5500**	**1.519**	**34.695**	**5.77**	**2.23**	**138.57**	**0.05**	**29.76**	**3.27×10^5**

***N.D., no data. T: Temperature, DO: dissolved oxygen.**

Table S2. The details of α-diversity indices for *Vibrio* community in each sample.

Station	Depth(m)	Shannon	Simpson	Chao 1	Sobs	Pielou
P1-19-1	5	1.837	0.720	73.111	67	0.303
	25	1.474	0.637	67	54	0.256
	105	0.777	0.320	28.333	25	0.167
	1000	1.291	0.579	35	20	0.299
	2000	0.946	0.511	46	24	0.206
	3000	0.840	0.510	6	5	0.362
	4000	3.040	0.895	81.333	81	0.480
	5500	1.112	0.617	19.333	16	0.278
P1-19-5	5	1.042	0.415	35.143	35	0.203
	55	1.857	0.769	43.333	40	0.349
	80	2.104	0.822	49.2	42	0.390
	155	2.268	0.863	81	36	0.439
	300	0.010	0.002	14.75	11	0.003
	700	1.24	0.554	141.12	113	0.186
	1000	1.285	0.664	288.75	63	0.215
	2000	1.369	0.622	19.5	18	0.328
	3000	0.364	0.105	177.6	116	0.053
	5000	0.208	0.058	130.588	87	0.032
	5900	2.599	0.870	78.6	65	0.432
P1-19-9	5	1.845	0.739	270.5	123	0.266
	55	1.506	0.575	154	80	0.238
	80	1.651	0.652	153.75	82	0.26
	155	1.123	0.549	101.526	87	0.174
	300	2.078	0.819	168.882	123	0.299

	700	2.230	0.863	43	29	0.459
	1000	1.437	0.722	32	28	0.299
	2000	0.234	0.07	43	38	0.045
	3000	0.542	0.203	63.273	55	0.094
	5000	0.282	0.084	50.091	46	0.051
	5750	1.638	0.799	14	13	0.443
P1-19-13	5	1.652	0.684	35	33	0.328
	55	1.86	0.784	25.5	25	0.4
	80	2.017	0.795	46.4293	46	0.365
	155	2.8	0.909	56.5	49	0.499
	300	0.273	0.09	25	24	0.06
	700	2.04	0.828	20	20	0.472
	1000	0.671	0.477	5	4	0.335
	2000	0.531	0.288	13	10	0.16
	3000	0.071	0.018	21	16	0.018
	4000	1.057	0.58	8	8	0.35
	5000	1.163	0.431	47	38	0.222
	6000	1.696	0.776	13	13	0.458
P1-19-21	5	1.675	0.736	28.333	28	0.348
	55	1.931	0.8	24.5	24	0.421
	105	1.691	0.679	26	25	0.364
	215	1.204	0.617	22	19	0.284
	500	1.678	0.767	19.6	19	0.395
	1000	0.399	0.123	167.474	103	0.06
	1500	0.434	0.143	116.792	92	0.067
	2800	0.98	0.451	15.5	14	0.257
P1-19-25	5	2.01	0.813	33.143	31	0.406

**55**	**1.255**	**0.59**	**25.5**	**24**	**0.274**
**80**	**1.056**	**0.386**	**35.5**	**33**	**0.209**
**105**	**1.904**	**0.731**	**47**	**40**	**0.358**
**155**	**1.427**	**0.71**	**31**	**19**	**0.336**
**300**	**0.859**	**0.321**	**47**	**26**	**0.183**
**500**	**1.639**	**0.71**	**39.5**	**29**	**0.337**
**700**	**1.796**	**0.769**	**20.5**	**20**	**0.415**
**1000**	**0.0089**	**0.002**	**12.333**	**9**	**0.002**
**2000**	**1.117**	**0.668**	**43**	**15**	**0.286**
**3000**	**0.912**	**0.53**	**28**	**25**	**0.196**
**5500**	**0.309**	**0.137**	**29.6**	**24**	**0.067**

Fig. S1 The vertical patterns for α-diversity indices of *Vibrio* community along the water depth.

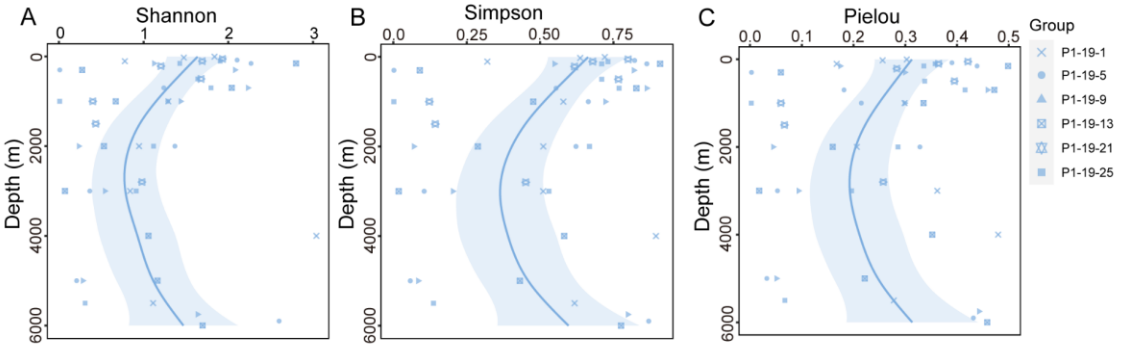

Supplement: Supplementary file 1 [file Data_Sheet_1.PDF]
